# iDPGK: characterization and identification of lysine phosphoglycerylation sites based on sequence-based features

**DOI:** 10.1186/s12859-020-03916-5

**Published:** 2020-12-09

**Authors:** Kai-Yao Huang, Fang-Yu Hung, Hui-Ju Kao, Hui-Hsuan Lau, Shun-Long Weng

**Affiliations:** 1 Department of Medical Research, Hsinchu Mackay Memorial Hospital, Hsinchu City 300, Taiwan; 2 Department of Medicine, Mackay Medical College, New Taipei City 252, Taiwan; 3 Department of Obstetrics and Gynecology, Hsinchu Mackay Memorial Hospital, Hsinchu City 300, Taiwan; 4 Department of Obstetrics and Gynecology, Mackay Memorial Hospital, Taipei City 104, Taiwan; 5 Mackay Junior College of Medicine, Medicine, Nursing and Management College, Taipei City 112, Taiwan

**Keywords:** Protein phosphoglycerylation, 3-Phosphoglyceryl-lysine (pgK), Post-translational modification (PTM), Sequence-based features

## Abstract

**Background:**

Protein phosphoglycerylation, the addition of a 1,3-bisphosphoglyceric acid (1,3-BPG) to a lysine residue of a protein and thus to form a 3-phosphoglyceryl-lysine, is a reversible and non-enzymatic post-translational modification (PTM) and plays a regulatory role in glucose metabolism and glycolytic process. As the number of experimentally verified phosphoglycerylated sites has increased significantly, statistical or machine learning methods are imperative for investigating the characteristics of phosphoglycerylation sites. Currently, research into phosphoglycerylation is very limited, and only a few resources are available for the computational identification of phosphoglycerylation sites.

**Result:**

We present a bioinformatics investigation of phosphoglycerylation sites based on sequence-based features. The TwoSampleLogo analysis reveals that the regions surrounding the phosphoglycerylation sites contain a high relatively of positively charged amino acids, especially in the upstream flanking region. Additionally, the non-polar and aliphatic amino acids are more abundant surrounding phosphoglycerylated lysine following the results of PTM-Logo, which may play a functional role in discriminating between phosphoglycerylation and non-phosphoglycerylation sites. Many types of features were adopted to build the prediction model on the training dataset, including amino acid composition, amino acid pair composition, positional weighted matrix and position-specific scoring matrix. Further, to improve the predictive power, numerous top features ranked by F-score were considered as the final combination for classification, and thus the predictive models were trained using DT, RF and SVM classifiers. Evaluation by five-fold cross-validation showed that the selected features was most effective in discriminating between phosphoglycerylated and non-phosphoglycerylated sites.

**Conclusion:**

The SVM model trained with the selected sequence-based features performed well, with a sensitivity of 77.5%, a specificity of 73.6%, an accuracy of 74.9%, and a Matthews Correlation Coefficient value of 0.49. Furthermore, the model also consistently provides the effective performance in independent testing set, yielding sensitivity of 75.7% and specificity of 64.9%. Finally, the model has been implemented as a web-based system, namely iDPGK, which is now freely available at http://mer.hc.mmh.org.tw/iDPGK/.

## Background

Protein post-translational modifications (PTMs) are generally enzymatic and covalent chemical modification of proteins following protein biosynthesis. Of the 20 amino acids that make up proteins, lysine is one of the most highly modified residues. According to the various studies reviewed, there are numerous common types of PTMs that occurred at lysine residues such as acetylation, ubiquitination, sumoylation, methylation, hydroxylation. These PTMs and enzymes are associated with a myriad of human diseases, including heart diseases, rheumatoid arthritis, multiple sclerosis, neurodegenerative diseases, celiac diseases and cancers.

Lysine phosphoglycerylation is a non-enzymatic PTM, which be identified in both human cells and mouse liver by Moellering and Cravatt [1], they found that phosphoglycerylation plays a key role in regulating glucose metabolism and glycolytic process. It exploits the electrophilicity of 1,3-bisphosphoglycerate (1,3-BPG) to modify specific lysine residues and thus form a 3-phosphoglyceryl-lysine (pgK) that function in glycolysis. A comprehensive proteomics analysis reveals that pgK-modified proteins create a potential feedback mechanism by inhibiting and accumulating glycolytic enzymes that leads to the accumulation of glycolytic intermediates to alternate biosynthetic pathways [2]. Furthermore, it has been demonstrated that abnormal phosphoglycerylation has a high chance to cause the congestive heart failure [3].

Due to the labile nature of PGK bond and the low abundance of endogenously phosphoglycerylated proteins in vivo, further research is needed to clarify the characteristics and mechanisms of lysine phosphoglycerylation. Although mass spectrometry has been available for detection of variety of PTMs in laboratories [4, 5], but there are still many deficiencies, the process is expensive, time-consuming and not as effective. Thus, it is necessary to develop a systematic method for identifying phosphoglycerylation sites of proteins in silico. As listed in Additional File 1: Table S1, Xu et al. [6] developed a computational analysis tool named Phogly-PseAAC evaluated using K-nearest neighbor (KNN) classifier and pseudo-amino acid composition to detect the phosphoglycerylation sites. Another prediction tool named CKSAAP_PhoglySite was developed to predict the phosphoglycerylation sites on human proteins using composition of k-spaced amino acid pairs (CKSAAP) and fuzzy support vector machine (SVM) with tenfold cross-validation, and they indicated that the effectiveness of predicted secondary structure features seems to have very little practical use for discriminating between phosphoglycerylation sites and non-phosphoglycerylation sites [7]. However, data size is a very crucial part of model training, more than total 2000-dimensional features was obtained by the CKSAAP encoding scheme which may cause overfitting with small sample size [8]. PhoglyPred is another predictor which focused on selecting the important sequence-based features using the F-score, and evaluated using SVM and jackknife test to predict the phosphoglycerylation sites; moreover, to improve the classification for the imbalanced dataset, the authors set the different parameters for positive and negative datasets [9]. Except for the sequence-based features, EvolStruct-Phogly has incorporated local structure conformations, accessible surface area (ASA) and position-specific scoring matrix (PSSM) to predict phosphoglycerylated lysine residues [10]. More recently, another prediction tool named Bigram-PGK which used evolutionary information in PSSM of protein sequences and its transformation to bigram occurrences appears to predict phosphoglycerylated sites [11]. Numerous analytical methods were proposed for predicting the phosphoglycerylation sites, which provide effective performance in cross-validation using training dataset. However, choosing the most reliable prediction method has been a challenge for researchers, because of there is a lack of independent testing to verify the objective effectiveness of these predictors.

In this study, we provide a full characterization of phosphoglycerylated substrate sites based on various features, including linear sequences and evolutionary information of amino acids. Subsequently, we build predictive models with both balance and imbalance datasets using decision tree (DT), random forest (RF) and support vector machine (SVM) algorithms. Furthermore, five-fold cross-validation was conducted to assess the effectiveness of the proposed models. Most important of all, an additional phosphoglycerylation dataset was divided from the raw dataset which completely blind to the training dataset, and an independent testing of state-of-the-art methods was performed on these data. To facilitate the study of protein lysine phosphoglycerylation, we are motivated to develop a web tool for the identification of phosphoglycerylation sites.

## Results

### Composition of amino acids around phosphoglycerylation sites

**Fig. 1:**
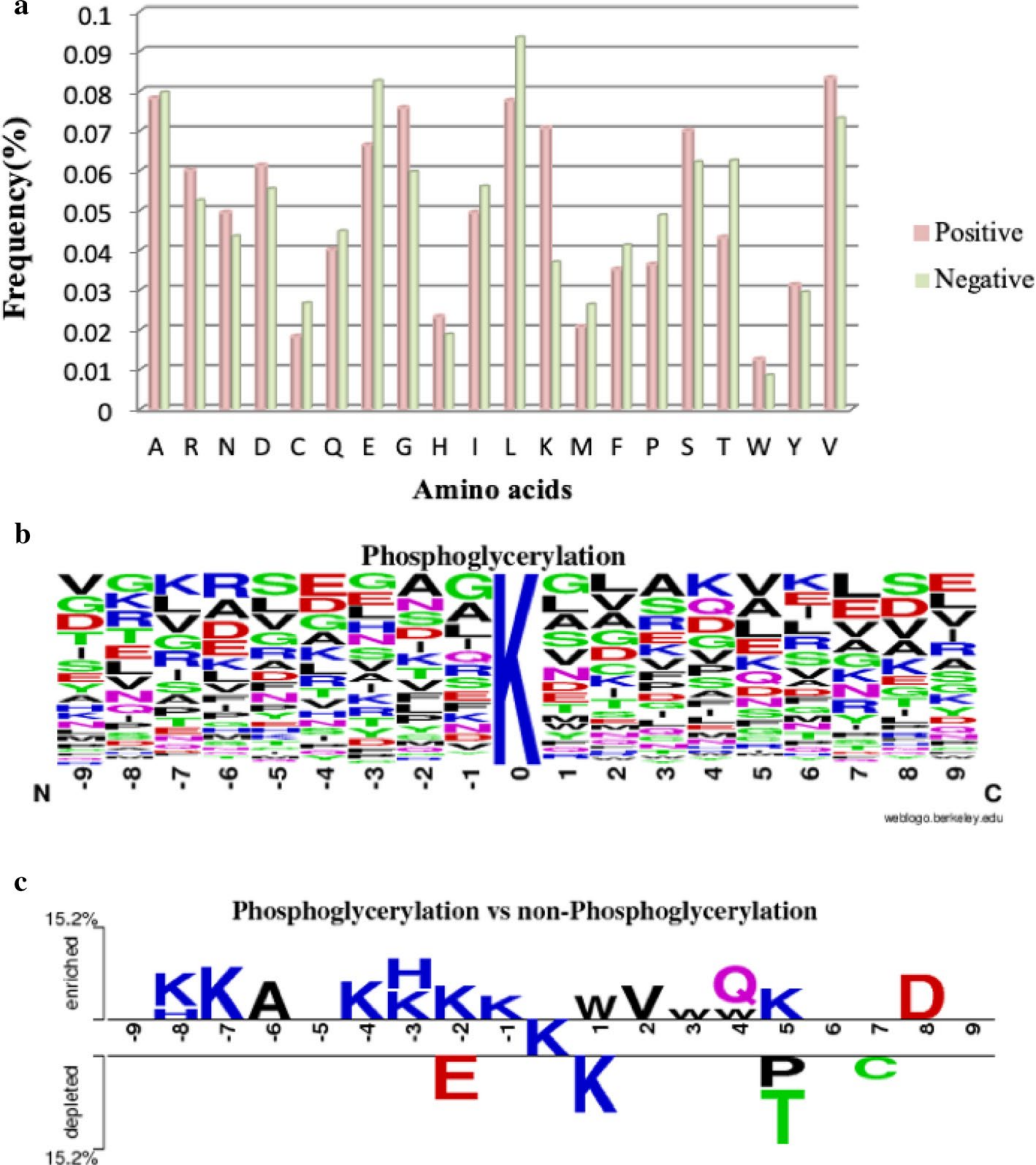
Composition of amino acids surrounding phosphoglycerylation sites. **a** Comparison of AAC between 89 positive and 178 negative sequences. **b** Position-specific AAC of 89 phosphoglycerylated fragment sequences. **c** Comparison of position-specific AAC between phosphoglycerylated and non-phosphoglycerylated sequences based on TwoSampleLogo analysis

In order to investigate the consensus motif surrounding phosphoglycerylated lysine residues, the frequency of occurrence around phosphoglycerylation sites of each of the 20 amino acids was measured based on a 19-mer window length, and the phosphoglycerylated lysine residue of each peptide was excluded from this calculation. Figure 1a indicates that, valine (V) residue occurs at a highest frequency surrounding the phosphoglycerylation sites; on the contrary, cystine (C) and tryptophan (W) which residues have the lowest frequencies. Comparison of the frequency of occurrence between phosphoglycerylation sites and non-phosphoglycerylation sites, for phosphoglycerylation sites, K residue has a relatively higher frequency, while aspartate (D), glycine (G), serine (S) and V residues also occur more frequently; in contrast, C, glutamate (E), leucine (L), proline (P) and threonine (T) have relatively fewer frequency. Furthermore, we performed a measurement of the position-specific amino acid composition surrounding the phosphoglycerylated sites based on the training dataset using WebLogo [12].

However, as shown in Fig. 1b, both of the frequency and entropy plots indicated that it is difficult to identify the phosphoglycerylated sites based on the position-specific residue composition. Thus, we utilized Two-SampleLogo tool [13] to identify the significance of enrichment or depletion in position-specific amino acid composition between phosphoglycerylated and non-phosphoglycerylated sites. A total of 89 phosphoglycerylated sites and 178 non-phosphoglycerylated sites were compared in Fig. 1c, it was realized that two positively amino acids K and H residues reach significant enrichment in the upstream flanking region (from positions − 1 to − 9), excepted at the position + 1. In particular, downstream on the peptide compared to the non-phosphoglycerylated site, the acidic amino acid D residue has the highest proportion at the position + 8 with *p* value < 0.01. On the contrary, for non-phosphoglycerylated sites, it shows that E is slightly more abundant at position − 2 that suggested a lack of negatively charged K, H and R residues closing to non-phosphoglycerylated sites.

**Fig. 2:**
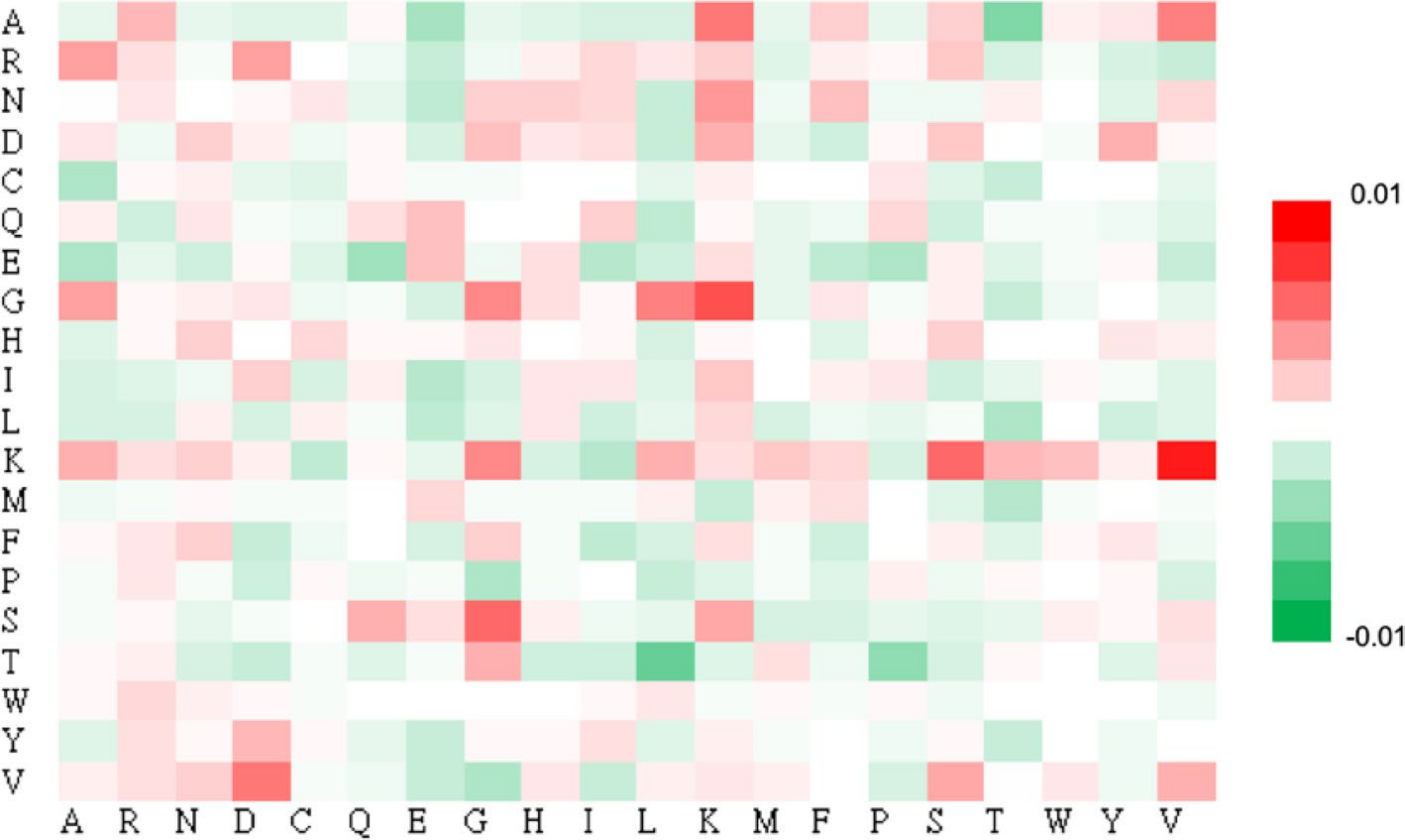
The frequency differences of 20 × 20 amino acid pairs between phosphoglycerylated sites and non-phosphoglycerylated sites

Besides composition of amino acids, the composition of amino acid pairs was also measured to explore the statistically significant dipeptides around phosphoglycerylation sites. As shown in Fig. 2, the over-represented amino acid pairs were highlighted in red color and the under-represented pairs were highlighted in green color by displaying in a 20 × 20 matrix. After ranking the amino acid pairs according to occurrence frequency, the dipeptides formation from K or G were found in the top ranking such as KV, AK, GL, GG and GK. This result indicated that most of the dipeptides involved the two residues are enriched surrounding the modified residues and were considered as statistically significant pairs for the identification of protein phosphoglycerylation sites.

### Identification of the sequence motifs based on position-specific background amino-acid probabilities

With the frequency plot of sequence logo representation given in Fig. 1b, there is no obvious feature representation for each position. To further investigate the potential phosphoglycerylation motif in primary sequence, we applied a program PTM-Logo [14] that identify the significantly enriched and underrepresented amino acids surrounding the phosphoglycerylation site based on the training dataset. The program makes appropriate adjustments to probabilities of amino acids at each position according to the PTM type and the positions relative to the modified site. But there is a limitation of length for input sequences, the maximum acceptable length of the program 15-mer (− 7, + 7) is selected as the window length in the following evaluation and implementation.

**Fig. 3:**
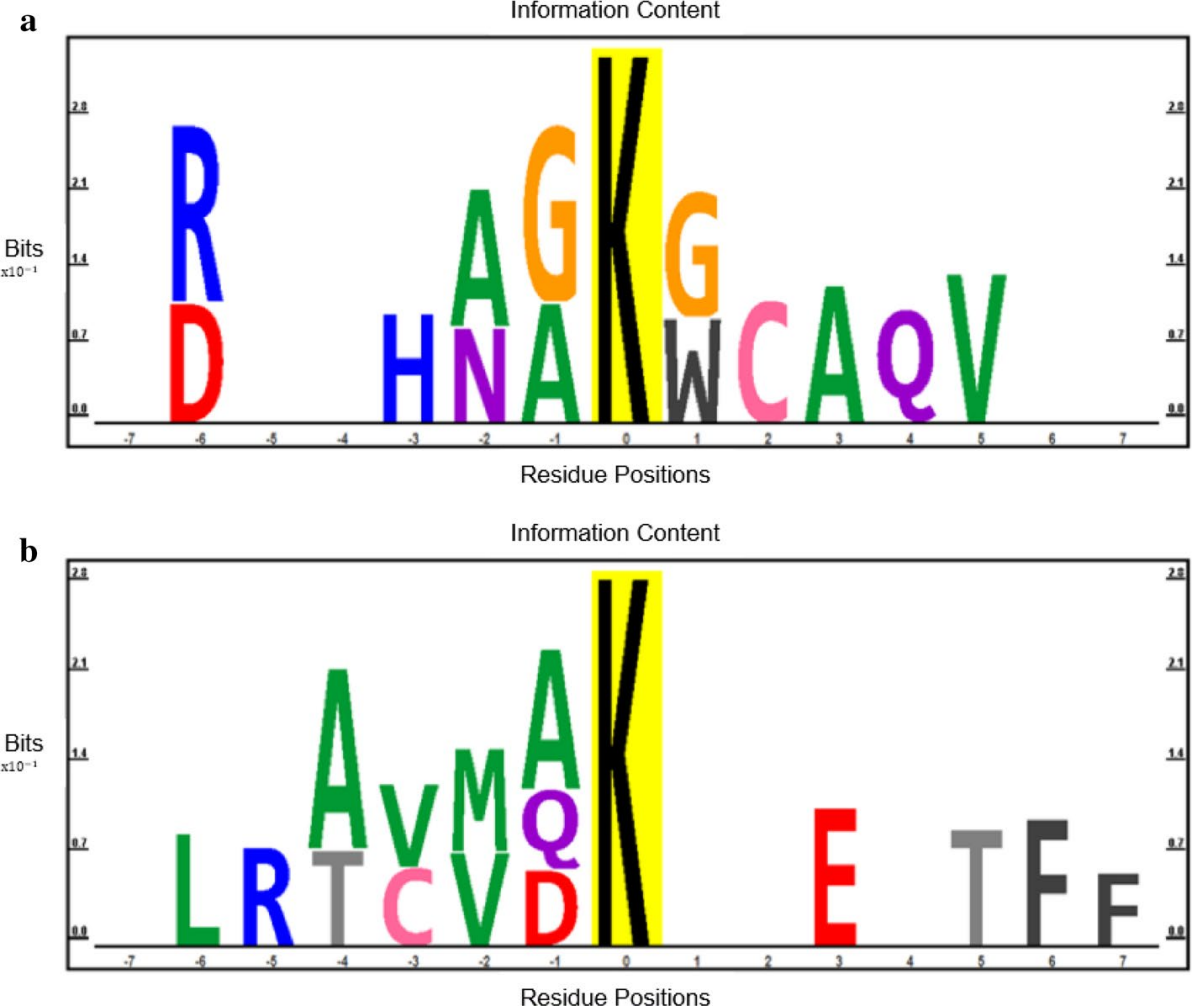
The motif analysis based on position-specific amino-acid probability backgrounds of 89 phosphoglycerylated sequences

Herein, Fig. 3a reveals that the one motif was detected based on the occurrence of R and D residues at upstream position − 6 of the peptide. It also shows that non-polar and aliphatic amino acids such as alanine (A), G, and V are more abundant surrounding phosphoglycerylated lysine at position − 2 to + 3 and 5. Position + 4 was exhibiting the highest proportion of polar amino acids namely glutamine (Q), and the positively charged and polar amino acid H had the highest ratios at position − 3. Additionally, the other motif was observed as shown in Fig. 3b, it was displaying the highest proportion of aromatic residues F (Phenylalanine) at position + 6 and + 7. Position − 5 was a special case, showing more abundant positively charged amino acid. The results indicated that the upstream region of the phosphoglycerylation sites harbor a notable abundance of positively charged amino acids, which is corresponding to the result of the TwoSampleLogo analysis. This analysis shows that, in a sequence, the amino acids with special properties surrounding PTM sites plays an important role in identifying the phosphoglycerylated sites.

### Cross-validation performance of the models trained with single type and multiple types of features

To determine sequence-based features can be adopted to distinguish between the phosphoglycerylation sites and non-phosphoglycerylation sites, various kinds of features were extracted to build the prediction models, including AAC, AAPC, BLOSUM62 and PSSM. In addition, the composition of positively charged amino acids (PCAAC) such as H, R and K residues, were especially extracted from AAC to build a prediction model. Each of above listed feature sets alone with the training dataset were fed into three different classification algorithms, such as support vector machine (SVM), random forest (RF) and decision tree (DT), and the models were evaluated using five-fold cross-validation. As shown in Table 1, the models were trained with single type of features using RF could provide the best overall performance in classifying between phosphoglycerylated and non-phosphoglycerylated lysine residues. The RF model trained with AAC has the great performance with a sensitivity of 59.6%, specificity of 59%, accuracy of 59.2%, and MCC value of 0.18, while that trained with AAPC gives a passable specificity of 62.9%. In particular, the SVM model trained with PCAAC alone provides the best overall performance comparing to the other models with a sensitivity of 58.4%, specificity of 68.0%, accuracy of 64.8%, MCC value of 0.25.

**Table 1:** Five-fold cross validation results of the DT, RF and SVM models trained on single type of features

Training feature	Classifier	Sensitivity (%)	Specificity (%)	Accuracy (%)	MCC
AAC	DT	59.6	55.6	56.9	0.14
	RF	59.6	59.0	59.2	0.18
	SVM	56.2	59.6	58.4	0.15
AAPC	DT	59.6	47.8	51.7	0.07
	RF	48.3	62.9	58.1	0.11
	SVM	60.7	47.8	52.1	0.08
B62	DT	44.9	70.8	62.2	0.16
	RF	55.1	55.1	55.1	0.10
	SVM	51.7	43.3	46.1	− 0.05
PSSM	DT	34.8	71.9	59.6	0.07
	RF	58.4	52.2	54.3	0.10
	SVM	39.3	59.6	52.8	− 0.01
PCAAC	DT	50.6	71.3	64.4	0.22
	RF	50.6	50.6	50.6	0.01
	SVM	58.4	68.0	64.8	0.25

**Fig. 4:**
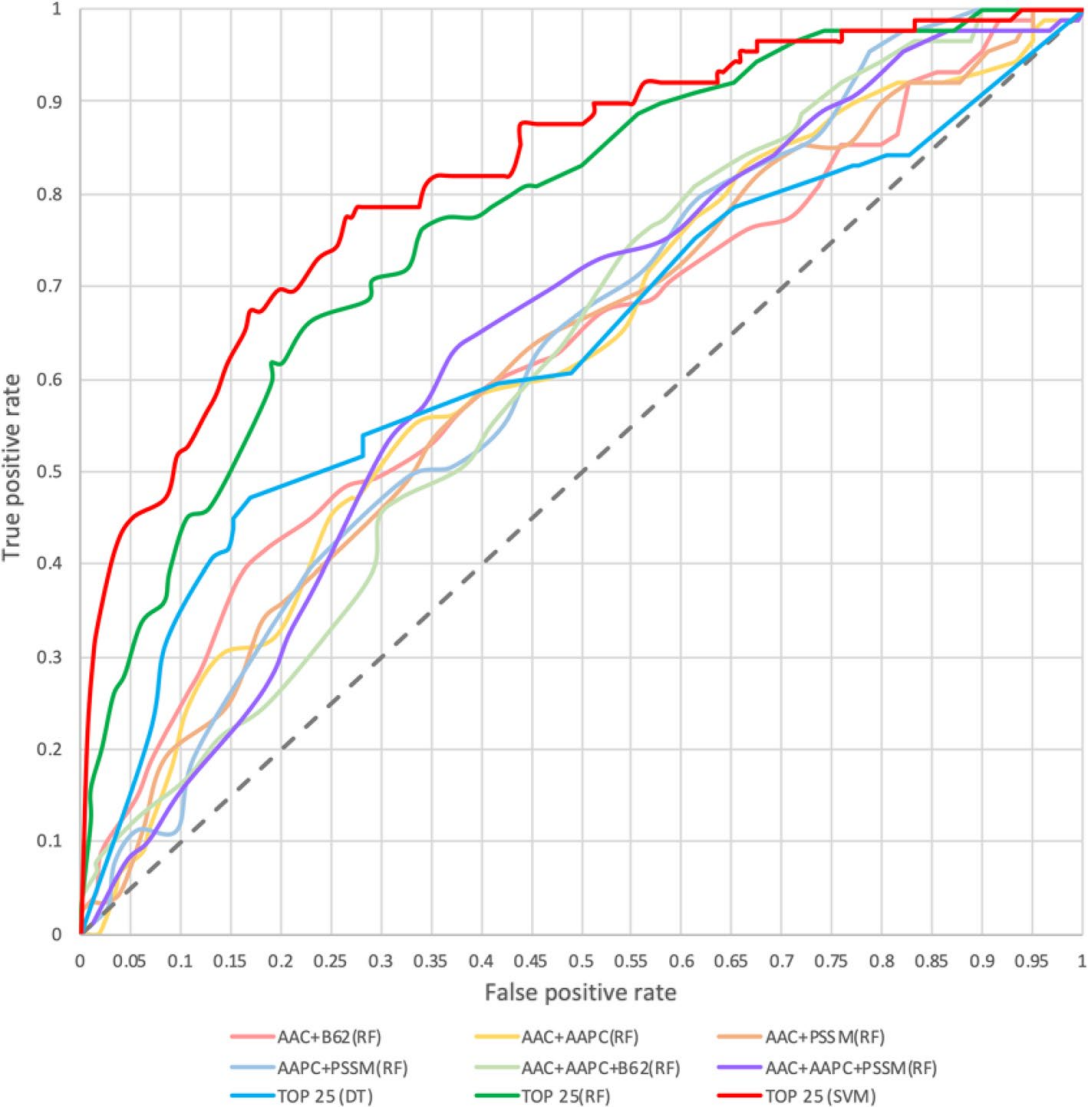
Comparison of ROC curves among the models trained using various features based on five-fold cross-validation

Moreover, to improve the predictive power, the hybrid models were trained by combining two or more different types of features that were also evaluated by five-fold cross-validation. Table 2 shows that comparing to the models trained with single type of features, the RF model trained with the combination of AAC, AAPC and PSSM features could significantly improve the performance with a sensitivity of 62.9%, a specificity of 62.9%, an accuracy of 62.9%, and the MCC value of 0.24. The models trained for each feature combination using RF still have the best performance when comparing to DT and SVM, thus Fig. 4 provides the comparison of ROC curves only among the models trained on different multiple types of features based on their five-fold cross-validation performance. According to the evaluation criteria, the model trained by combining AAC, AAPC and PSSM using RF classifier exhibited the best overall performance among various predictive models.

**Table 2:** Five-fold cross validation results of the DT, RF and SVM models trained with multiple types of features

Training feature	Classifier	Sensitivity (%)	Specificity (%)	Accuracy (%)	MCC
AAC + AAPC	DT	53.9	50.6	51.7	0.04
	RF	58.4	60.1	59.6	0.18
	SVM	53.9	57.3	56.2	0.11
AAC + B62	DT	42.7	66.3	58.4	0.09
	RF	59.6	59.0	59.2	0.18
	SVM	68.5	34.8	46.1	0.03
AAC + PSSM	DT	32.6	64.6	53.9	− 0.03
	RF	59.6	59.0	59.2	0.18
	SVM	39.3	59.6	52.8	− 0.01
AAPC + PSSM	DT	31.5	69.7	56.9	0.01
	RF	62.9	54.5	57.3	0.16
	SVM	39.3	59.6	52.8	− 0.01
AAC + AAPC + B62	DT	40.4	63.5	55.8	0.04
	RF	60.7	54.5	56.6	0.14
	SVM	68.5	34.3	45.7	0.03
AAC + AAPC + PSSM	DT	31.5	64.6	53.6	− 0.04
	RF	62.9	62.9	62.9	0.24
	SVM	69.7	39.3	49.4	0.09

### Performance evaluation of the models trained with the selected sequence-based features

Based on the cross-validation results presented above, there is a significant difference in the predictive performance between the models trained with the same features but using different classifiers. In this study, no matter which features are used, the models trained using RF classifier that provide the better performance compared to others. Notable, according to the random forest algorithm, a random subset of the features was selected at each candidate split in the learning process, it means that the performance was not influenced by all the features. Therefore, in order to investigate the selected sequence-based features, a total of 4 kinds of features were ranked by F-score, including AAC, AAPC, B62 and PSSM. Subsequently, according to the process of forward feature selection, sequentially add only one attribute into the model to evaluate the performance at each step, continuing until the integration of features could not further improve the predictive performance.

And lastly, a total of 25 selected attributes were considered as the final combination for further classification as listed in Additional file 2: Table S2, the predictive models trained on these crucial features using DT, RF and SVM classifiers, the performance of each model was also evaluated by five-fold cross-validation as presented in Table 3, and the ROC curves of the models also shown in Fig. 4. The results show that the RF model could provide a better performance than previous ones, which has the sensitivity of 70.8%, the specificity of 70.8%, the accuracy of 70.8% and the MCC value of 0.40. In a surprise twist, the SVM model gave the best predictive performance comparing to DT and RF models, which could reach the sensitivity of 77.5%, the specificity of 73.6%, the accuracy of 74.9 and the MCC value of 0.49. In summary, the SVM model trained with the top 25 features selected by F-score and SFS can significantly enhance the performance of the model for predicting the protein phosphoglycerylation sites.

**Table 3:** Five-fold cross validation results of the DT, RF and SVM models trained with the selected features

Classifier	Sensitivity (%)	Specificity (%)	Accuracy (%)	MCC
DT	59.6	58.4	58.8	0.17
RF	70.8	70.8	70.8	0.40
SVM	77.5	73.6	74.9	0.49

### Implementation of web-based tool for identifying phosphoglycerylation sites

Given a protein sequence, tandem mass spectrometry is the main technology currently used for identification of the post-translational modified sites [15]; however, the researchers still encounter equipment and technical difficulties such expensive, time-consuming and labor-intensive process. Thus, an effective prediction tool should be developed to efficiently identify potential phosphoglycerylation sites. In this work, the SVM model trained with the selected sequence-based features is utilized to develop a web-based online tool for automatic prediction of phosphoglycerylation sites, named iDPGK. The system allows users to submit the protein sequences of interest in FASTA format, which efficiently returns the predictive results including the potential position of phosphoglycerylated sites and the bar plot for amino acid composition surrounding the modified residue. To demonstrate the performance of iDPGK, an experimentally-verified phosphoglycerylated proteins are used as case studies which are not included in the training data set. The human gamma-enolase (ENOG_HUMAN) contains one verified phosphoglycerylation site at Lys-351, out of 23 lysine residues in the protein sequence, iDPGK could make an accurate prediction for the only one validated site.

## Discussion

In classifying between the phosphoglycerylation and non-phosphoglycerylation sites, the model trained on the training dataset might be overestimated, which could lead to the overfitting problem. Thus, an independent testing dataset of phosphoglycerylation sites was split from the non-homologous dataset and used to verify the predictive power of the final model, which consisted of 37 positive sites and 74 negative sites.

As given in Table 4, the SVM model constructed with the top 25 selected features provides 75.7%, 64.9%, 70.3% and 0.41 for sensitivity, specificity, accuracy and MCC value, respectively. In summary, based upon independent testing, the result shows that the proposed SVM model can outperform other models in overall and can handle class imbalance in classification between phosphoglycerylation sites and non-phosphoglycerylation sites.

**Table 4:** Comparison of independent testing results between our method and the available prediction tools

Classifier	Sensitivity (%)	Specificity (%)	Accuracy (%)	MCC
Phogly-PseAAC	59.5	67.4	67.2	0.09
iPGK-PseAAC	37.8	96.2	94.5	0.27
iDPGK (our method)	75.7	64.9	70.3	0.41

## Conclusion

This study contributes to providing a comprehensive characterization of phosphoglycerylation sites based on sequence analysis of the experimentally verified modified sites due to the lack of experimentally determined phosphoglycerylation protein structures. Through observation of the results of WebLogo and TwoSampleLogo, the analysis of position-specific amino acids composition between phosphoglycerylation and non-phosphoglycerylation site reveals that the regions surrounding the modified sites contain a high relatively of positively charged amino acids, especially in the upstream flanking region. Additionally, the non-polar and aliphatic amino acids are more abundant surrounding phosphoglycerylated lysine following the results of PTM-Logo. As stated previously, these investigations suggested that the composition of amino acids can play a crucial role in distinguishing between phosphoglycerylation and non-phosphoglycerylation sites. In summary, based on the results of this study, it is suggested that the phosphoglycerylation sites generally occur not only within a positively charged region but also within a conserved motif. According to the evaluation by five-fold cross-validation, the SVM model was trained on all the training data using the top 25 sequence-based features ranked by F-score measurements, which was used to predict the phosphoglycerylation sites.

**Fig. 5:**
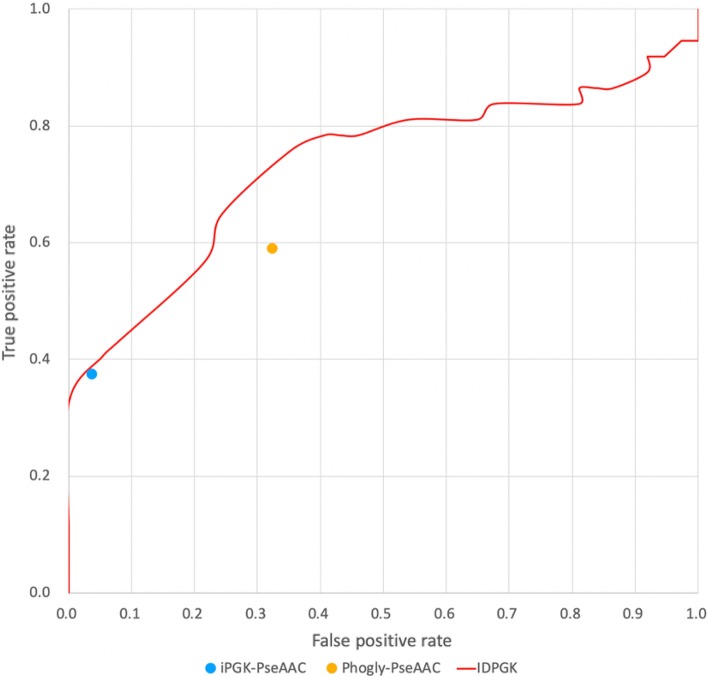
Comparison of the predictive performance between the proposed models and existing prediction tools based upon independent testing

Moreover, to further demonstrate the effectiveness of the proposed model, a comparison between our model and previous models using the independent test dataset is given. Considering previously published studies, there are only two existing prediction tools, namely Phogly-PseAAC [6] and iPGK-PseAAC [16]. The results, as shown in Table 3, Phogly-PseAAC provided a sensitivity of 59.5%, a specificity of 67.4%, an accuracy of 67.2%, and an MCC of 0.09. Meanwhile, the iPGK-PseAAC provides 37.8%, 96.2%, 94.5% and 0.27 for sensitivity, specificity, accuracy and MCC value, respectively. The independent testing demonstrated that iDPGK provided a better predictive performance with balanced sensitivity and specificity, 75.7% and 64.9%, respectively. Moreover, as shown Fig. 5, the ROC curve displays that our model yielded a slightly higher true positive rate (sensitivity) when at the same level of false positive rate (1-specificity) for each tool. Consequently, the proposed model was employed to develop a web-based tool to identify phosphoglycerylation sites based on sequence-based features, named iDPGK (http://mer.hc.mmh.org.tw/iDPGK). Most important of all, with the availability of data increasing rapidly, the proposed method is applicable for analysis of the large-scale proteomics dataset with no adjustment required.

## Methods

### Data collection and pre-processing

In this study, the phosphoglycerylated substrate sites were collected from Protein Lysine Modification Database (PLMD) [17], a manually curated database of experimentally verified lysine modification sites which contains 187 phosphoglycerylation sites of 137 proteins. Lysine phosphoglycerylation is a recent research area in proteomics, the data are quite limited at present; therefore, the collected data were randomly split into two sets for model training (150 sites) and independent testing (37 sites) with the conventional ratio of 8:2. The testing dataset was used to evaluate the state-of-art prediction tools, which were compared with the presented method in terms of predictive performance.

**Fig. 6:**
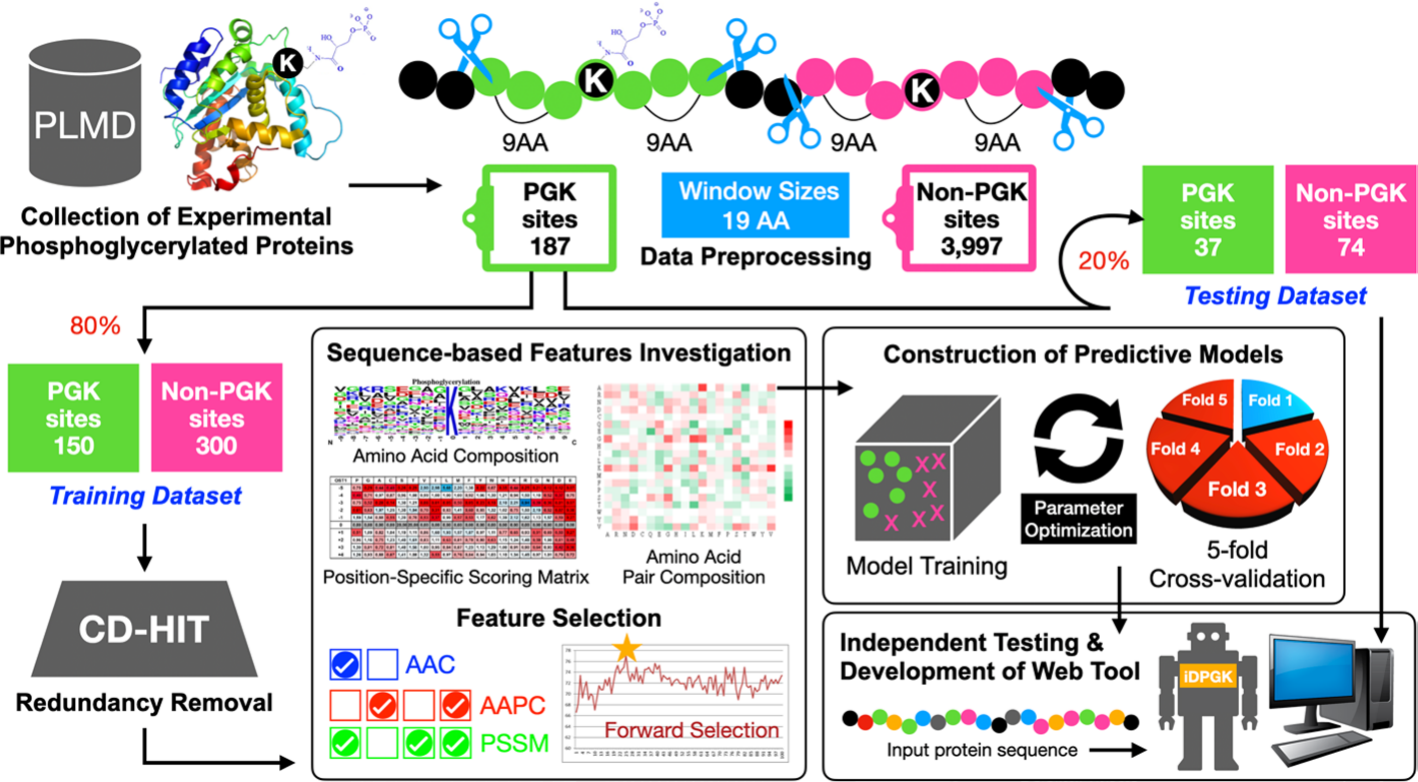
The analytical flowchart of the identification of protein phosphoglycerylation sites

The analytical flowchart of this work is described in Fig. 6. With reference to our previous work [18], sequence fragments with a window length of 2n + 1 centering at the experimentally verified phosphoglycerylation sites were extracted as the positive dataset; besides, the lysine residues without annotation on the phosphoglycerylated proteins that these fragments were extracted as the negative dataset. To determine an appropriate window size for model construction, we performed the evaluations of the models under the different window lengths on the basis of SVM classifier with amino acid composition features. As the results of five-fold cross validation, the model trained using 19-mer window length could achieve the best accuracy as shown in Additional File 1: Table S1.

In this work, to elude the overestimation of predictive performance, CD-HIT program [19] was used to remove homologous sequences from the training dataset with 40% sequence identity. Considering the limited positive data availability, the negative datasets were randomly extracted from the corresponding original datasets with the ratio of 1:2 between the number of positive and negative sequences. As shown in Table 5, 178 non-phosphoglycerylation sites were randomly selected for training dataset and 74 for independent testing dataset, respectively.

**Table 5:** Data statistics of training and testing datasets after the removal of homologous sequences using CD-HIT program

Sequence identity cut-off	Number of phosphoglycerylation sites	Number of non-phosphoglycerylation sites
Raw data	150	3997
90%	107	3031
80%	104	2610
70%	98	2319
60%	96	2040
50%	93	1845
40%	89	1318
Training data	89	178
Independent testing data	37	74

### Features extraction and encoding

After the sequence extraction process, we focused on the analysis of sequence-based features, and then each sequence fragment was encoded based on the investigated features. The following sequence-based features are widely employed for analysis and prediction of various types of PTM sites in the enormous amount of research [18, 20, 21]: amino acid composition (AAC), positively charged amino acid composition (PCAAC), amino acid pair composition (AAPC), BLOSUM62 scoring matrix (B62) and position-specific scoring matrix (PSSM). In this study, the phosphoglycerylated sequences should be transformed into numeric vectors based on the above features to construct a supervised learning model.

The composition of amino acid (AAC) is a widely-used feature for calculating the frequencies of each amino acid in a given protein sequence [22]. There are 21 types of amino acids that need to be considered for feature encoding, including 20 native and 1 unnatural amino acid. The composition of amino acid pairs (AAPC) is another sequence based feature introduced by Park and Kanehisa [23], transforms a sequence fragment into a 441-dimensional vector, which includes 441 elements specifying the numbers of occurrences of amino acid dipeptides normalized with the total number of dipeptides in a sequence fragment. The scoring matrix for amino acid substitutions, also known as BLOSUM62 (B62) matrix, was built based on the frequencies of amino acid substitutions in clusters of proteins that with less than 62% identity between two sequences. With reference to our previous work [24], each fragment was represented by a matrix of (2n + 1) × *w* elements, where 2*n* + 1 represents the length of the sequence fragment and *w* stands for 21 elements including 20 types of amino acids and one for the non-existing residue. Position-specific scoring matrix (PSSM) is a matrix which contains the evolutionary information of considered proteins calculated from the probability matrix and the background probabilities. In this work, the PSSM profiles of each phosphoglycerylated protein were derived by using PSI-BLAST search against the non-redundant database of protein sequences from NCBI [25, 26]. The matrix consists of (2*n* + 1) × 20 elements where 2*n* + 1 represents the length of the sequence fragment and 20 stands for the sums of position specific scores for each type of amino acid.

In order to investigate the important features for the prediction of protein phosphoglycerylation sites, the predictive power of each feature attribute is evaluated on the training data based on cross-validation. Additionally, to obtain the highest predictive accuracy, the hybridized-feature vectors were combined for improving predictive performance on the classification between phosphoglycerylated and non-phosphoglycerylated sites.

### Selection of the best hybrid feature sets

F-score is most typically used for feature selection, which is defined as the weighted harmonic mean of both the precision and the recall of the test [27]. There is an 842-dimensional feature vector made up of sequential and statistical features, which was composed by three types of features including AAC, AAPC and PSSM. By referring to the CNN-SuccSite method [20], all the features were sorted and ranked according to F-score on training dataset prior to construction of predictive models. Furthermore, the sequential forward selection (SFS) [28] is a type of stepwise regression which involves beginning with an empty model and testing the addition of each variable, then adding the variables one at a time until none improves the model to a statistically significant extent. Finally, we determined the final combination of hybrid feature sets using SFS based on the F-score ranking results.

### Construction of predictive models and performance measurement

In this study, the training dataset was composed of 89 phosphoglycerylation sites and 178 non-phosphoglycerylation sites, which used for model construction by using WEKA software. Based on the binary classification, there were three types of learning algorithms such as LIBSVM [29], Random Forests (RF) [30] and Decision Tree (DT) [31], which were used to build the predictive models for discriminating the phosphoglycerylation sites from non-phosphoglycerylation sites. For the LIBSVM classifier, the radial basis function (RBF) was adopted as the kernel function, which determined by a gamma parameter while the cost parameter was used to modulate the softness of the hyper-plane [29]. Random forests (RF) is an ensemble learning method for classification and regression by combining multitude of decision trees, and each tree depends on the values of a random feature sets sampled independently [30]. Random forest is then considered as an appropriate classifier to handle moderately imbalanced dataset refer to previous study [32]. Decision tree (DT) is a tree-like model in which each internal node represents a “test” on an attribute, each branch represents the outcome of the test, and each leaf node represents a class label [31]. J48 is a Java implementation of C4.5 decision tree algorithm integrated in WEKA software, the constructed decision tree was used as the model for classification.

To avoid overfitting, five-fold cross-validation was organized to examine the capability of the investigated features in classification between phosphoglycerylation sites and non-phosphoglycerylation sites, which was carried out for each feature set to evaluate the predictive performance. The training dataset was randomly split into five subgroups, the model was trained using 4 of the subgroups and the resulting model was validated on the remaining part of the data, and tests each subset only once, and then the process was repeated five times. The performance measure reported by five-fold cross-validation is then the average of the values computed in the loop. To estimate the predictive performance of the model, the following measures were used, sensitivity (Sn), specificity (Sp), accuracy (Acc), and Matthews Correlation Coefficient (MCC):

$$\begin{aligned} & Sensitivity = \frac{TP}{{TP + FN}} \\ & Specificity = \frac{TN}{{TN + FP}} \\ & Accuracy = \frac{TP + TN}{{TP + FP + TN + FN}} \\ & {\text{MCC}} = \frac{{\left( {TP \times TN} \right) - \left( {FP \times FN} \right)}}{{\sqrt {\left( {TP + FP} \right)\left( {TP + FN} \right)\left( {TN + FP} \right)\left( {TN + FN} \right)} }} \\ \end{aligned}$$

## Supplementary Information

**Additional file 1**. **Table S1.** Determining the window size for further analysis based on the five-fold cross validation results of the SVM models trained on amino acid composition.

**Additional file 2**. **Table S2.** List of the top 25 features ranking by F-score and the sequential forward selection method.

## Data Availability

The iDPGK tool and datasets used in this work can be accessed at the following URL: http://mer.hc.mmh.org.tw/iDPGK/.

## Abbreviations

PGK: 3-Phosphoglyceryl-lysine
AAC: Amino acid composition
AAPC: Amino acid pair composition
B62: BLOSUM62 scoring matrix
PSSM: Position-specific scoring matrix
PCAAC: Positively charged amino acid composition
Acc: Accuracy
MCC: Matthews correlation coefficient
PLMD: Protein lysine modifications database
PTM: Post-translational modification
RBF: Radial basis function
Sn: Sensitivity
Sp: Specificity
SVM: Support vector machine
RF: Random forests
DT: Decision tree
SFS: Sequential forward selection
