# Supplementary material for: iDPGK: characterization and identification of lysine phosphoglycerylation sites based on sequence-based features
Source: BMC Bioinformatics. 2020 Dec 9;21:568. doi: 10.1186/s12859-020-03916-5 (PMC7727188; doi:10.1186/s12859-020-03916-5)
Supplement: Supplementary file 1 — Additional file 1. Table S1. Determining the window size for further analysis based on the five-fold cross validation results of the SVM models trained on amino acid composition. [file 12859_2020_3916_MOESM1_ESM.pdf]

Table S1. Determining the window size for further analysis based on the five-fold cross validation results of the SVM models trained on amino acid composition.

Window sizes	TP	FP	TN	FN	Sensitivity	Specificity	Accuracy	MCC
11	39	42	40	43	47.6%	48.8%	48.2%	-0.04
13	41	38	49	46	47.1%	56.3%	51.7%	0.03
15	41	43	36	38	51.9%	45.6%	48.7%	-0.03
17	46	42	42	38	54.8%	50.0%	52.4%	0.05
19	61	34	55	28	68.5%	61.8%	65.2%	0.30
21	47	33	54	40	54.0%	62.1%	58.0%	0.16
23	47	39	47	39	54.7%	54.7%	54.7%	0.09
25	45	37	46	38	54.2%	55.4%	54.8%	0.10
27	47	40	42	35	57.3%	51.2%	54.3%	0.09
29	56	37	45	26	68.3%	54.9%	61.6%	0.23
31	48	40	42	34	58.5%	51.2%	54.9%	0.10
