# Supplementary material for: iDPGK: characterization and identification of lysine phosphoglycerylation sites based on sequence-based features
Source: BMC Bioinformatics. 2020 Dec 9;21:568. doi: 10.1186/s12859-020-03916-5 (PMC7727188; doi:10.1186/s12859-020-03916-5)
Supplement: Supplementary file 2 — Additional file 2. Table S2. List of the top 25 features ranking by F-score and the sequential forward selection method. [file 12859_2020_3916_MOESM2_ESM.pdf]

Table S2. List of the top 25 features ranking by F-score and the sequential forward selection method.

Rank	Category	Feature	F-score
1	AAC	K	0.092040
2	AAPC	VD	0.055901
3	AAPC	KV	0.042421
4	PSSM	Q at +1 position	0.040316
5	AAPC	KW	0.039683
6	AAPC	SG	0.039635
7	PSSM	K at -8 position	0.038410
8	PSSM	P at +2 position	0.031342
9	AAPC	TL	0.030303
10	PSSM	R at -8 position	0.028557
11	AAC	T	0.027611
12	PSSM	I at -5 position	0.027576
13	AAPC	YD	0.026379
14	AAPC	GL	0.026112
15	PSSM	P at -5 position	0.026062
16	AAPC	AT	0.026018
17	PSSM	C at 0 position	0.025288
18	PSSM	K at +5 position	0.025076
19	PSSM	A at -3 position	0.025057
20	AAPC	DY	0.024728
21	PSSM	M at +3 position	0.024603
22	AAPC	GK	0.023979
23	PSSM	C at -8 position	0.023399
24	AAPC	WR	0.023256
25	AAPC	KS	0.022738

*AAC, Amino acid composition; AAPC, Amino acid pair composition; PSSM, Position-specific scoring matrix.
